# Panax Quinquefolius Saponin of Stem and Leaf Attenuates Intermittent High Glucose-Induced Oxidative Stress Injury in Cultured Human Umbilical Vein Endothelial Cells via PI3K/Akt/GSK-3****β**** Pathway

**DOI:** 10.1155/2013/196283

**Published:** 2013-07-15

**Authors:** Jingshang Wang, Huijun Yin, Ye Huang, Chunyu Guo, Chengdong Xia, Qian Liu, Lu Zhang

**Affiliations:** ^1^Department of Cardiovascular Disease, Xiyuan Hospital, China Academy of Chinese Medical Sciences, Beijing 100091, China; ^2^Department of Endocrinology, Xiyuan Hospital, China Academy of Chinese Medical Sciences, Beijing 100091, China

## Abstract

Panax quinquefolius saponin of stem and leaf (PQS), the effective parts of American ginseng, is widely used in China as a folk medicine for diabetes and cardiovascular diseases treatment. In our previous studies, we have demonstrated that PQS could improve the endothelial function of type II diabetes mellitus (T2DM) rats with high glucose fluctuation. In the present study, we investigated the protective effects of PQS against intermittent high glucose-induced oxidative damage on human umbilical vein endothelial cells (HUVECs) and the role of phosphatidylinositol 3-kinase kinase (PI3K)/Akt/GSK-3**β** pathway involved. Our results suggested that exposure of HUVECs to a high glucose concentration for 8 days showed a great decrease in cell viability accompanied by marked MDA content increase and SOD activity decrease. Moreover, high glucose significantly reduced the phosphorylation of Akt and GSK-3**β**. More importantly, these effects were even more evident in intermittent high glucose condition. PQS treatment significantly attenuated intermittent high glucose-induced oxidative damage on HUVECs and meanwhile increased cell viability and phosphorylation of Akt and GSK-3**β** of HUVECs. Interestingly, all these reverse effects of PQS on intermittent high glucose-cultured HUVECs were inhibited by PI3K inhibitor LY294002. These findings suggest that PQS attenuates intermittent-high-glucose-induced oxidative stress injury in HUVECs by PI3K/Akt/GSK-3**β** pathway.

## 1. Introduction

Vascular disorders, especially cardiovascular disorders, are major causes of morbidity and mortality in diabetic patients [1]. Intermittent high glucose (IHG) and constant high glucose are two general phenomena in diabetes. Recent studies have shown that IHG may be more dangerous for the development of diabetes-related complications including cardiovascular disorders, and thus for diabetic patients [2]. 

Although the precise mechanism underlying the action of IHG remains unclear, significant progress has been made. Recent studies have shown that IHG could induce an increased rate of apoptosis, protein kinase C activation, nicotinamide adenine dinucleotide phosphate oxidase activation in cultured endothelial cells, and monocytes adhesion to endothelial cells in diabetic rats. These effects are even more pronounced than those of constant high glucose [3–7]. Moreover, there is growing evidence that an acute increase of glycemia is accompanied by oxidative stress that may contribute to the generation of vascular endothelial dysfunction [8]. Meanwhile, clinical evidence suggests that *in vivo* glucose fluctuations may be damaged for endothelial cells, which could be mediated by oxidative stress [9, 10]. And enhanced oxidative damage after diverse stimuli has been confirmed to be an initial event in the development of cardiovascular diseases [11, 12]. It is, therefore, thought that prevention of intermittent high glucose-induced oxidative damage on endothelial cells may have important implications for pharmacologic attempts to prevent these complications.

PQS is the effective parts of American ginseng, a herb widely used in clinical Chinese medicine for diabetes and cardiovascular diseases treatment. In fact, there is growing evidence demonstrating the significant beneficial effects of PQS consumption on diabetic patients, including lowering blood sugar, keeping blood sugar stable, improving insulin resistance, regulating lipid metabolism, and cardioprotective effects such as antimyocardial ischemia, reducing myocardium oxygen consumption, ameliorating myocardial remodeling, and inhibiting platelet aggregation [13–16]. A multicenter, double-blind, randomized control clinical trial organized by Ministry of Public Health of the People's Republic of China showed the similar results [17]. Our previous study had demonstrated that PQS could improve endothelial function in diabetes mellitus in experimental rats with high glucose fluctuation [18]. However, as far as we know, little evidence exists concerning the effect of PQS on oxidative damage in endothelial cells induced by intermittent high glucose.

PI3K and Akt are downstream effectors of insulin signaling [19], as well as important signaling molecules in the regulation of glycogen metabolism in myocytes, lipocytes, and hepatocytes [20, 21]. Uncoupling of insulin signaling at PI3K-Akt in response to high glucose concentrations in these cell types has been implicated in the pathogenesis of insulin resistance and T2DM [22]. By regulating angiogenesis, proliferation, microvascular permeability, survival, cellular transformation, and embryonic differentiation, PI3K-Akt also plays an important role in regulation of endothelial cell (EC) function [23, 24]. Cells respond via PI3K-Akt signaling to a variety of cytokines, G protein-coupled receptor ligands, and growth factors as well as to cellular stresses, including heat shock, hypoxia, and oxidative stress [25]. It has been reported that prolonged exposure of ECs to high glucose concentrations would result in reduced proliferation and survival through altered PI3K-Akt signaling [26]. The nitric oxide production, followed by Akt activation, had been confirmed to prevent steady high blood glucose-induced endothelial cell injury [27]. Our previous study had observed that PQS could improve insulin sensitivity by increasing the tyrosine phosphorylation of insulin receptor and IRS1 and the Ser473 phosphorylation of Akt [15]. A potential target of PQS may also be the serine/threonine kinase Akt.

Therefore, the aims of our present study were to (1) determine whether treatment with PQS attenuates intermittent high glucose-induced stress injury in HUVECs and, if so, (2) investigate the signaling pathway involved.

## 2. Materials and Methods

### 2.1. Chemicals and Reagents

PQS was provided by Jilin Jian Yisheng Pharmaceutical Co. Ltd. Dulbecco's-Modified Eagle's Medium (DMEM) was purchased from Gibco (Grand Island, NY, USA). Fetal bovine serum (FBS) was obtained from HyClone. The antibodies against Akt, phosphorylated-Akt (Ser473), GSK3*β*, GSK3*β*, phosphorylated-GSK3*β* (Ser9) and *β*-Actin were purchased from Cell Signaling Technology (USA). The LY294002 and 3-(4, 5-dimethylthiazol-2-yl) 2, 5-diphenyltetrazolium bromide (MTT) were purchased from Sigma-Aldrich (St. Louis, USA). Malonyldialdehyde (MDA) and superoxide dismutase (SOD) assay kits were obtained from Jian Cheng Biological Engineering Institute (Nanjing, China). All other biochemicals used were of the highest purity available. 

### 2.2. Isolation and Culture of Human Umbilical Vein Endothelial Cells (HUVECs)

**Figure 1:**
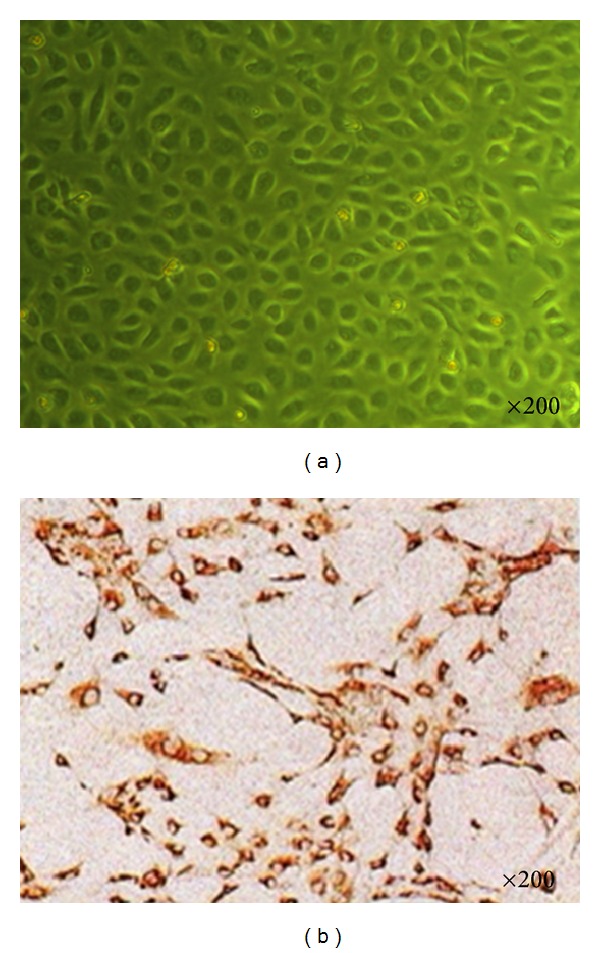
Morphology and immunocytochemical staining of HUVEC. (a) Characteristic cobblestone morphology of HUVEC under an inverted microscope. (b) Immunocytochemical staining of HUVEC.

HUVECs were isolated and pooled from umbilical cords obtained from normal vaginal deliveries by the procedure described by Jaffe et al. [28]. The cells were cultured in gelatin-coated 60 mm Petri dishes (Corning) and grown in DMEM, supplemented with 20% heat-inactivated fetal bovine serum, 20 mM glutamine (Sigma-Aldrich), 10 ng/mL endothelial cell growth supplement (Sigma-Aldrich), 40 U/mL heparin (Gibco), 50 U/mL penicillin, 50 *μ*g/mL streptomycin (Gibco), 20 mM Hepes (Sigma-Aldrich), and 0.11 mg/mL sodium pyruvate (Sigma-Aldrich). The Petri dishes were incubated at 37°C, in 5% CO_2_-95% air gas mixture. Primary cultures were fluid-changed 24 h after seeding and were subcultured on reaching confluence by the use of 0.25% Trypsin-EDTA, inactivated by dilution. More than 99% HUVECs were identified to be endothelial cells by their characteristic cobblestone morphology (Figure 1(a)) under an inverted microscope (Leica DMIRB, Germany) and characterized by brown granules in cytoplasm using immunocytochemical staining of factor VIII (Figure 1(b)). Only HUVECs of the second passage were used in the study to avoid age-dependent cellular modification. HUVECs were seeded at equal density in gelatin-coated 60 mm Petri dishes or plates, allowed to attach overnight, and then exposed to the following experimental conditions for 8 days: (1) continuous DMEM containing normal (5.56 mmol/L) glucose (NG), (2) continuous DMEM containing high (25 mmol/L) glucose (HG), (3) alternating normal and high-glucose media every 24 h (IHG), (4) as (3), with the addition of 0.05 mg/mL PQS, and (5) as (3), with the addition of 0.1 mg/mL PQS. To further examine the role of the PI3K/Akt/GSK3*β* pathway on the effect of PQS, another two groups of HUVECs were pretreated with the specific PI3K inhibitor LY294002 (20 *μ*mol/L; Sigma) for 30 min before PQS was added.

### 2.3. Cell Viability Measurement (MTT Assay)

Cell viability was determined by MTT assay. HUVECs were seeded in 96-well culture plates at a density of 1 × 10^5^ cells with 200 *μ*L culture medium per well. Four hour before the culture was terminated, 10 *μ*L assay medium containing 5 mg/mL MTT was added to each well. After 4 h of incubation at 37°C, the medium was removed and the cells lysed by addition of 150 *μ*L DMSO. The optical density of each sample was measured in an ELISA microplate reader using test and reference wavelengths of 490 nm.

### 2.4. Preparation of Cell Lysates

The cells were seeded at a density of 1 × 10^5^ cells/mL in 24-well plates and were allowed to attach for 24 h before treatment. Upon completion of the incubation studies, the cells were scraped from the plates into ice-cold RIPA lysis buffer (50 mM Tris with pH 7.4, 150 mM NaCl, 1% Triton X-100, 1% sodium deoxycholate, 0.1% SDS, and 0.05 mM EDTA), and protein concentration was determined by the bicinchoninic acid method, using BSA as a reference standard. Aliquots were stored at −80°C until detection for MDA and SOD activity.

### 2.5. Assay for Intracellular Contents of SOD and MDA

The activities of SOD and the concentration of MDA were both determined by using commercially available kits. All procedures completely complied with the manufacturer's instructions. The activities of SOD were expressed as units per milligram protein. MDA was measured at a wavelength of 532 nm by reacting with thiobarbituric acid to form a stable chromophoric production. Values of MDA level were expressed as nanomoles per milligram protein.

### 2.6. Western Blot Analysis

Cells were lysed in iced lysis buffer. Total protein (50 mg/lane) was separated by SDS-PAGE and transferred to a polyvinylidene fluoride membrane. After incubation in blocking solution (5% nonfat milk) (Sigma), membranes were incubated with primary antibodies for Akt, phosphorylated-Akt, GSK3*β*, phosphorylated-GSK3*β*, or *β*-Actin for overnight at 4°C. Membranes were washed and then incubated with 1 : 2000 dilution horseradish peroxidase-conjugated secondary antibody (ZSGB-BIO, Beijing, China). The relative density of each protein band was normalized to that of *β*-Actin. All results were representative of at least 3 independent experiments.

### 2.7. Statistical Analysis

All data were expressed as mean ± SD. The SPSS Statistics 15.0 package was utilized to analyze the data. Differences among groups were analyzed using the one-way analysis of variance (ANOVA), followed by multiple comparisons by LSD test. The *P* < 0.05 was considered statistically significant.

## 3. Results

### 3.1. Effects of PQS on Intermittent High Glucose-Induced Loss of HUVEC Viability

**Figure 2:**
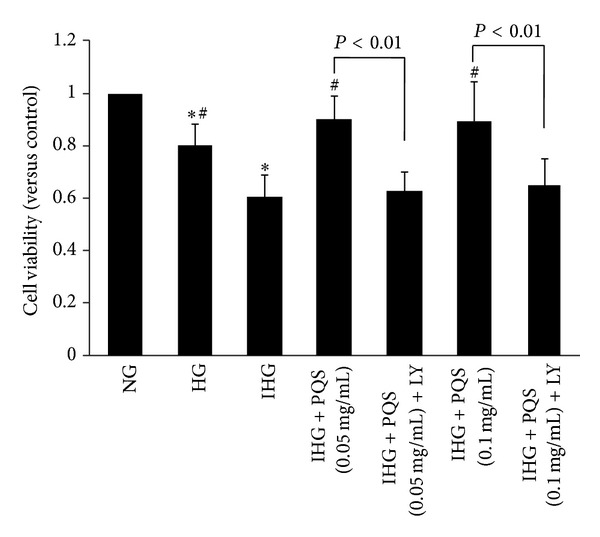
Effects of PQS on intermittent high glucose-induced loss of HUVEC viability. Cell viability was determined by MTT assay. Cell viability was determined by MTT assay. Cell viability was expressed as a percentage of cytoprotection versus control group set at 100%. Data were presented as means ± SD. (*n* = 5). *P < 0.01 versus NG; ^#^ *P* < 0.01 versus IHG.

After 8 days of experiment, we observed that the cell viability in HG (0.8 ± 0.12) decreased significantly in comparison with NG, and this decrease was even more marked in IHG (0.61 ± 0.08). Two different concentrations (0.05 or 0.1 mg/mL) of PQS improved cell viability significantly (0.9 ± 0.11 or 0.89 ± 0.15). However, pretreatment with LY294002 (PI3K inhibitor) abolished PQS's effect on cell viability in cultured endothelial cells exposed to intermittent high glucose (0.63 ± 0.07 or 0.65 ± 0.13) (Figure 2).

### 3.2. Effects of PQS on SOD and MDA Levels

**Figure 3:**
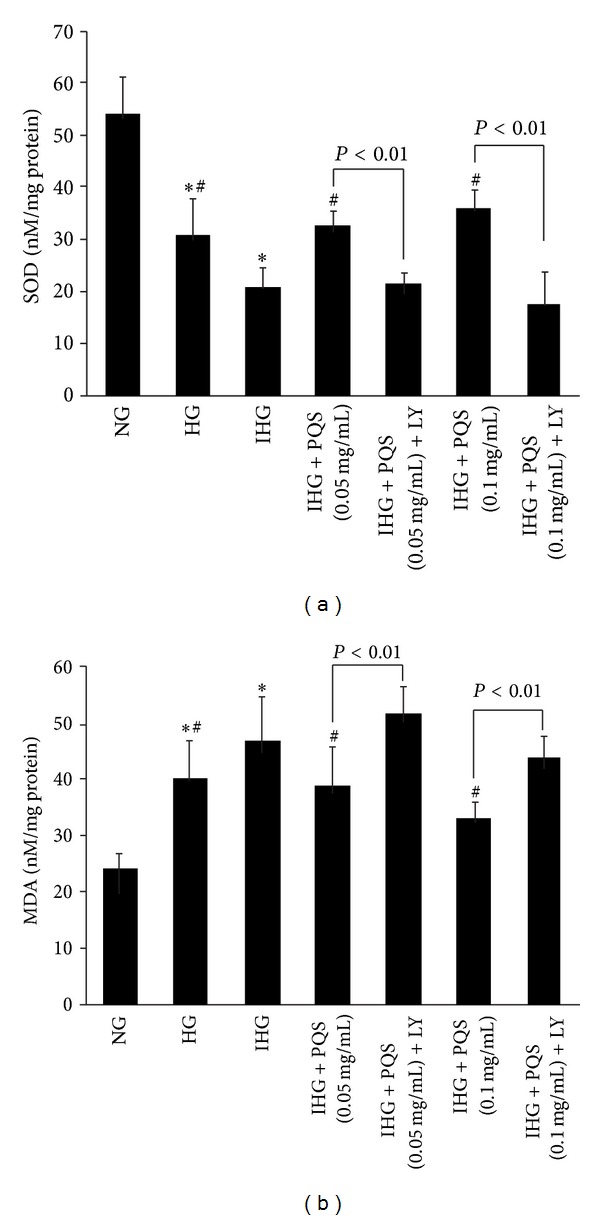
Effects of PQS on SOD (a) and MDA (b) content in HUVECs exposed to intermittent high glucose. Data were presented as means ± SD. (*n* = 5). *P < 0.01 versus NG, ^#^ *P* < 0.01 versus IHG.

As shown in Table 1 and Figure 3(a), after 8 days of experiment, the SOD level significantly decreased in IHG compared with either NG or HG. Pretreatment HUVECs with PQS (0.05 mg/mL or 0.1 mg/mL) inhibited the decreased SOD level induced by intermittent high glucose, which was abrogated by LY294002.

The content of MDA in the medium was increased significantly after treatment with intermittent high glucose for 48 h, compared with either normal or stable high glucose condition. Pretreatment HUVECs with PQS (0.05 mg/mL or 0.1 mg/mL) inhibited the elevation of MDA concentration elicited by intermittent high glucose significantly, which was abolished by LY294002 (Table 1 and Figure 3(b)).

Together, these results showed that blood glucose fluctuation produced higher suppression of antioxidant capacity and more oxidative damage than stable high glucose alone. However, pretreated with PQS, all these were reversed.

### 3.3. Effects of PQS on Decreased Akt and GSK3*β* Phosphorylation Levels Induced by Intermittent High Glucose

**Figure 4:**
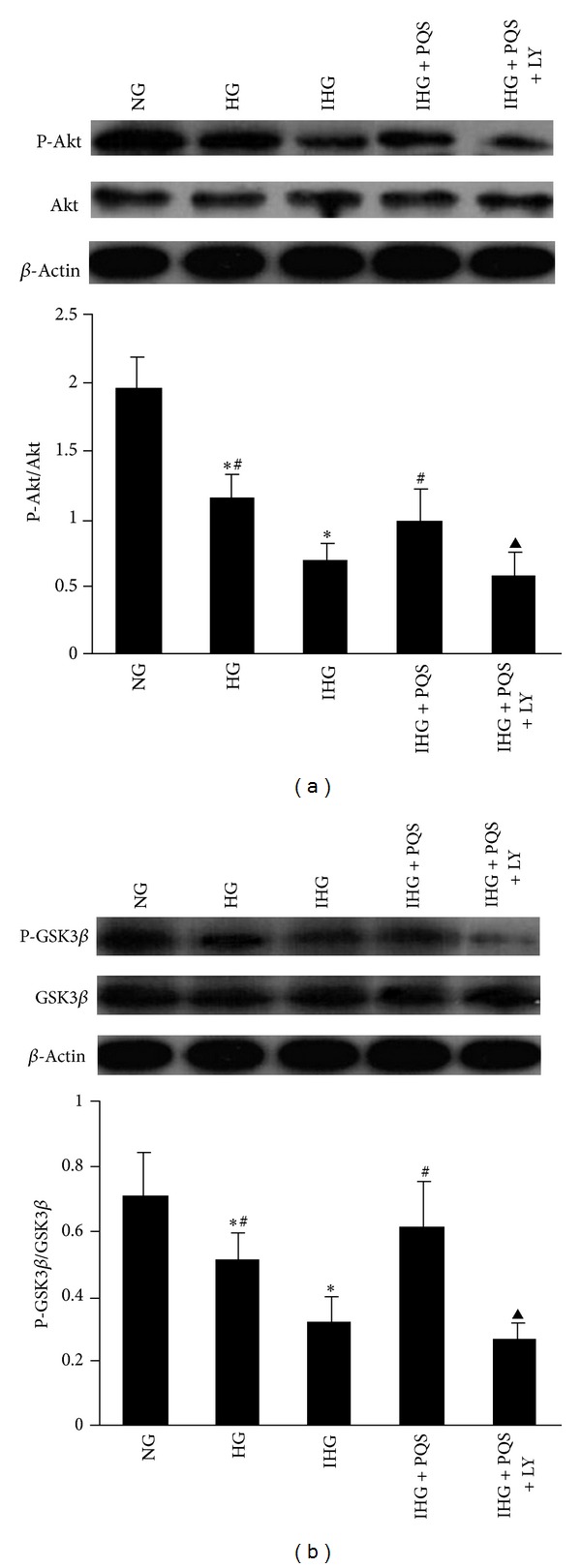
Phosphorylation of Akt (a) and GSK3*β* (b) in cultured human umbilical vein endothelial cells determined by western blot. Data obtained from quantitative densitometry were presented as mean ± SD. (*n* = 3). Before PQS was added, HUVECs were pretreated with LY294002 for 30 min. *P < 0.01 versus NG, ^#^ *P* < 0.01 versus IHG; ^▲^ *P* < 0.01 versus IHG + PQS. PQS, panax quinquefolius saponin of stem and leaf (0.1 mg/mL).

To investigate the underlying mechanism for protective effects of PQS, we examined the effect of PQS (0.1 mg/mL) on Akt and GSK3*β* level in intermittent high glucose-treated HUVECs. As shown in Figure 4(a), HG significantly reduced the phosphorylation of Akt without alteration of total Akt expression in comparison with conditioning, and this decrease was even more marked in IHG. Pretreatment of HUVECs with PQS led to a significant increase in the phosphorylation of Akt in endothelial cells exposed to intermittent high glucose. And PQS had no effect on the Akt protein level. The specific PI3K inhibitor LY294002 markedly suppressed the effects of PQS on Akt activity.

Furthermore, similar to the effects of HG/IHG and PQS on Akt phosphorylation, IHG inhibited GSK3*β* phosphorylation without alteration of total GSK3*β* expression, compared with NG or HG. PQS treatment significantly attenuated the decreased phosphorylation of GSK3*β* induced by intermittent high glucose, which was abolished by LY294002 (Figure 4(b)).

## 4. Discussion

There are two novel observations in our present experiment. Firstly, we have provided direct *in vitro* evidence that treatment with PQS attenuates intermittent high glucose-induced oxidative stress injury in HUVECs. Secondly, we have demonstrated that the protective effect of PQS on HUVECs was PI3K/Akt/GSK3*β*-dependent.

Hyperglycemia is generally regarded as one of the major causes of pathological consequences of both type I and type II diabetes [29]. Much of this damage is thought to be a consequence of elevated production of ROS and oxidative stress has recently been proposed as the unifying explanation of the hyperglycemia-related diabetic complications [30, 31]. In normal subjects, blood glucose is strictly controlled within a narrow range, while blood glucose in diabetic patients often changes obviously within a single day. Though there is still an extensive debate about glucose variability as a risk factor for complications independent of HbA1c in diabetes [32, 33], more and more lines of evidence have found that glucose fluctuations may play a significant role in the pathogenesis of diabetic complications. According to *in vitro* experimental settings and animal studies, fluctuating glucose levels display a more deleterious effect on endothelial cells than constantly high glucose exposure and that this effect should be mediated by an oxidative stress [3–7]. Moreover, human studies have shown that acute and chronic blood glucose fluctuations in T2DM levels could increase oxidative stress significantly [9, 10], although short-term glucose variability is not associated with raised oxidative stress markers in healthy volunteers [34].

In this study, we employed a cellular experimental model in which primary cultures of human endothelial cells were exposed to intermittent high glucose, a condition that partly mimics glucose excursions occurring in diabetes *in vivo*. As known to all, MDA is a by-product of lipid peroxidation induced by excessive ROS and widely used as a biomarker of oxidative stress [35]. On the other hand, SOD, as an endogenous antioxidant, plays a pivotal role in preventing cellular damage caused by ROS [36]. Enhanced oxidative damage after diverse stimuli has been confirmed to be an initial event in the development of cardiovascular diseases. In agreement with previous studies [6, 7], we confirmed that intermittent high glucose, as seen in diabetic patients, was more deleterious than those of stable high glucose and that oxidative stress was convincingly involved. In our present study, a more obvious decrease of cell viability was observed in HUVECs exposed to intermittent high glucose for 8 days, which was associated with an elevation of MDA production and a significant decrease in SOD. Nonetheless, when HUVECs were preincubated with PQS, these intermittent high glucose-induced cellular events were blocked to a great extent. These results together suggested that enhancement of endogenous antioxidant preservation and attenuation of lipid peroxidation may represent a major mechanism of cellular protection by PQS.

The underlying mechanism by which PQS protects HUVECs from intermittent high glucose-induced oxidative damage is an important question raised by the results presented in this study. 

Akt, downstream of PI3K, is thought to be one of the important factors in cell survival. In endothelial cells, Akt activation has been reported to promote cell survival [37, 38]. And evidence has shown that the PI3K/Akt pathway plays an important role in preventing endothelial cell injury induced by high glucose. Our previous study had confirmed that PQS could increase insulin sensitivity by increasing the tyrosine phosphorylation of insulin receptor and IRS1 and the Ser473 phosphorylation of Akt. Based on these observations, we examined the contribution of the PI3K/Akt pathway to the protective effect of PQS. In the present study, we confirmed the inhibitory effect of high glucose on Akt activation as previous report [39] and meanwhile observed a more obvious inhibitory effect in intermittent high glucose condition. We also demonstrated that PQS treatment attenuated the decrease in Akt phosphorylation induced by intermittent high glucose, which was abolished by PI3K inhibitor. Furthermore, LY294002 significantly abolished the protective effect of PQS on oxidative damage induced by intermittent high glucose, which indicated that PQS exerted its protective effect through PI3K/Akt pathway.

Among the various intracellular downstream effectors of Akt, GSK-3*β* phosphorylation and inactivation are considered important mechanisms of cell survival [40]. In the present study, we confirmed for the first time that decreased GSK3*β* phosphorylation level was involved in high glucose-induced oxidative damage. And the decrease was even more obvious in intermittent high glucose condition. Pretreatment with PQS significantly improved the decreased GSK3*β* phosphorylation levels induced by intermittent high glucose, which was also blocked by LY294002, indicating that PQS-promoted GSK3*β* phosphorylation depends on PI3K-Akt activation. Taken together, these results provide strong evidence that the PI3K/Akt/GSK3*β* pathway is involved in the antioxidative damage effect of PQS.

In summary, the present study shows that PQS inhibits intermittent high glucose-induced oxidative damage in cultured HUVECs through the PI3K/Akt/GSK3*β* pathway. It provides further strong evidence that PQS, as well as traditional Chinese herb, might offer an alternative strategy for diabetic cardiovascular complications prevention.

## Figures and Tables

**Table 1:** Effects of PQS on SOD and MDA levels (*n* = 5).

Group	SOD U/mg protein	MDA nmol/mg protein
NG	53.8 ± 7.62	24 ± 2.41
HG	30.81 ± 6.97^∗#^	39.9 ± 7.18^∗#^
IHG	20.73 ± 3.75*	47.16 ± 7.77*
IHG + PQS (0.05 mg/mL)	32.69 ± 2.66^#^	39.15 ± 6.86^#^
IHG + PQS (0.05 mg/mL) + LY	21.41 ± 2.05^▲^	52 ± 4.72^▲^
IHG + PQS (0.1 mg/mL)	35.9 ± 3.37^#^	33.11 ± 3.07^#^
IHG + PQS (0.1 mg/mL) + LY	17.66 ± 5.93^▲^	44.2 ± 3.66^▲^

Note: *P < 0.01 versus NG; ^#^ *P* < 0.01 versus IHG. ^▲^ *P* < 0.01 versus IHG + PQS (0.05 mg/mL or 0.1 mg/mL).

## References

[1] Ali MK, Narayan KMV, Tandon N (2010). Diabetes & coronary heart disease: current perspectives. *Indian Journal of Medical Research*.

[2] Brownlee M, Hirsch IB (2006). Glycemic variability: a hemoglobin A1c-independent risk factor for diabetic complications. *Journal of the American Medical Association*.

[3] Watada H, Azuma K, Kawamori R (2007). Glucose fluctuation on the progression of diabetic macroangiopathy-new findings from monocyte adhesion to endothelial cells. *Diabetes Research and Clinical Practice*.

[4] Mita T, Otsuka A, Azuma K (2007). Swings in blood glucose levels accelerate atherogenesis in apolipoprotein E-deficient mice. *Biochemical and Biophysical Research Communications*.

[5] Piconi L, Quagliaro L, Da Ros R (2004). Intermittent high glucose enhances ICAM-1, VCAM-1, E-selectin and interleukin-6 expression in human umbilical endothelial cells in culture: the role of poly(ADP-ribose) polymerase. *Journal of Thrombosis and Haemostasis*.

[6] Piconi L, Quagliaro L, Assaloni R (2006). Constant and intermittent high glucose enhances endothelial cell apoptosis through mitochondrial superoxide overproduction. *Diabetes/Metabolism Research and Reviews*.

[7] Horváth EM, Benko R, Kiss L (2009). Rapid “glycaemic swings” induce nitrosative stress, activate poly(ADP-ribose) polymerase and impair endothelial function in a rat model of diabetes mellitus. *Diabetologia*.

[8] Giacco F, Brownlee M (2010). Oxidative stress and diabetic complications. *Circulation Research*.

[9] Chang C-M, Hsieh C-J, Huang J-C, Huang I-C (2012). Acute and chronic fluctuations in blood glucose levels can increase oxidative stress in type 2 diabetes mellitus. *Acta Diabetologica*.

[10] Monnier L, Mas E, Ginet C (2006). Activation of oxidative stress by acute glucose fluctuations compared with sustained chronic hyperglycemia in patients with type 2 diabetes. *Journal of the American Medical Association*.

[11] Lakshmi SVV, Padmaja G, Kuppusamy P, Kutala VK (2009). Oxidative stress in cardiovascular disease. *Indian Journal of Biochemistry and Biophysics*.

[12] Elahi MM, Kong YX, Matata BM (2009). Oxidative stress as a mediator of cardiovascular disease. *Oxidative Medicine and Cellular Longevity*.

[13] Yin H-J, Zhang Y, Jiang Y-R (2004). The effect of panax quinquefolium saponins on blood lipid level in Alloxan-Induced hyperglycemia rat model. *Chinese Journal of Integrative Medicine on Cardio/Cerebrovascular Disease*.

[14] Yin H-J, Zhang Y, Yang L-H, Bai G-R, Shi D-Z, Chen K-J (2007). The effects of PQS on glucose transport, GLUT4 translocation and CAP mRNA expression of adipocytes. *Chinese Pharmacological Bulletin*.

[15] Zhang Y, Chen KJ, Yang L-H (2010). Effects of panax quinquefolius saponin of stem and leaf on glucose-lipid metabolism and insulin signal transduction in insulin resistant model adipocytes. *Zhongguo Zhong Xi Yi Jie He Za Zhi*.

[16] Fan B-J, Fei F, Zhao X-Z (2009). Effects of panax quinquefolium saponin (PQS) on the vascular endothelial function of the myocardial hypertrophied rats. *Chinese Journal of Gerontology*.

[17] Zhang Y (2006). *Effect of Panax Quinquefolius Saponin on Insulin Sensitivity in Patients of Coronary Heart Disease and Its Mechanism*.

[18] Wang J-S, Yin H-J, Guo C-Y (2013). Influence of high blood glucose fluctuation on the endothelial function of type 2 diabetes mellitus rats and the effects of panax quinquefolius saponin of stem and leaf. *Chinese Journal of Integrative Medicine*.

[19] Galetic. I, Andjelkovic. M, Meier R, Brodbeck D, Park J, Hemmings BA (1999). Mechanism of protein Kinase B activation by insulin/Insulin-like growth Factor-1 revealed by specific inhibitors of phosphoinositide 3-kinase-significance for diabetes and cancer. *Pharmacology and Therapeutics*.

[20] Hernandez R, Teruel T, Lorenzo M (2001). Akt mediates insulin induction of glucose uptake and up-regulation of GLUT4 gene expression in brown adipocytes. *FEBS Letters*.

[21] Tremblay F, Lavigne C, Jacques H, Marette A (2001). Defective insulin-induced GLUT4 translocation in skeletal muscle of high fat-fed rats is associated with alterations in both Akt/protein kinase B and atypical protein kinase C (zeta/lambda) activities. *Diabetes*.

[22] Vosseller K, Wells L, Lane MD, Hart GW (2002). Elevated nucleocytoplasmic glycosylation by O-GlcNAc results in insulin resistance associated with defects in Akt activation in 3T3-L1 adipocytes. *Proceedings of the National Academy of Sciences of the United States of America*.

[23] Gousseva N, Kugathasan K, Chesterman CN, Khachigian LM (2001). Early growth response factor-1 mediates insulin-inducible vascular endothelial cell proliferation and regrowth after injury. *Journal of Cellular Biochemistry*.

[24] Shioi T, McMullen JR, Kang PM (2002). Akt/protein kinase B promotes organ growth in transgenic mice. *Molecular and Cellular Biology*.

[25] De Luca A, Maiello MR, D’Alessio A, Pergameno M, Normanno N (2012). The RAS/RAF/MEK/ERK and the PI3K/AKT signalling pathways: role in cancer pathogenesis and implications for therapeutic approaches. *Expert Opinion on Therapeutic Targets*.

[26] Varma S, Lal BK, Zheng R (2005). Hyperglycemia alters PI3k and Akt signaling and leads to endothelial cell proliferative dysfunction. *American Journal of Physiology*.

[27] Zhang W, Wang R, Han S-F (2007). *α*-Linolenic acid attenuates high glucose-induced apoptosis in cultured human umbilical vein endothelial cells via PI3K/Akt/eNOS pathway. *Nutrition*.

[28] Jaffe EA, Nachman RL, Becker CG, Minick CR (1973). Culture of human endothelial cells derived from umbilical veins. Identification by morphologic and immunologic criteria. *The Journal of Clinical Investigation*.

[29] Shamoon H, Duffy H, Fleischer N (1993). The effect of intensive treatment of diabetes on the development and progression of long-term complications in insulin-dependent diabetes mellitus. *The New England Journal of Medicine*.

[30] Ceriello A, Esposito K, Piconi L (2008). Oscillating glucose is more deleterious to endothelial function and oxidative stress than mean glucose in normal and type 2 diabetic patients. *Diabetes*.

[31] Brownlee M (2005). The pathobiology of diabetic complications: a unifying mechanism. *Diabetes*.

[32] Siegelaar SE, Holleman F, Hoekstra JBL, DeVries JH (2010). Glucose variability; does it matter?. *Endocrine Reviews*.

[33] Kilpatrick ES, Rigby AS, Atkin SL (2010). Glucose variability and diabetes complication risk: we need to know the answer. *Diabetic Medicine*.

[34] Wakil A, Smith KA, Atkin SL, Kilpatrick ES (2012). Short-term glucose variability in healthy volunteers is not associated with raised oxidative stress markers. *Diabetes, Obesity & Metabolism*.

[35] Cini M, Fariello RG, Bianchetti A, Moretti A (1994). Studies on lipid peroxidation in the rat brain. *Neurochemical Research*.

[36] Luo T, Xia Z (2006). A small dose of hydrogen peroxide enhances tumor necrosis factor-alpha toxicity in inducing human vascular endothelial cell apoptosis: reversal with propofol. *Anesthesia and Analgesia*.

[37] Kim I, Kim H-G, So J-N, Kim JH, Kwak HJ, Koh GY (2000). Angiopoietin-1 regulates endothelial cell survival through the phosphatidylinositol 3’-kinase/Akt signal transduction pathway. *Circulation Research*.

[38] Fulton D, Gratton JP, McCabe TJ (1999). Regulation of endothelium-derived nitric oxide production by the protein kinase Akt. *Nature*.

[39] Ho FM, Lin WW, Chen BC (2006). High glucose-induced apoptosis in human vascular endothelial cells is mediated through NF-*κ*B and c-Jun NH2-terminal kinase pathway and prevented by PI3K/Akt/eNOS pathway. *Cellular Signalling*.

[40] Park K-W, Yang H-M, Youn S-W (2003). Constitutively active glycogen synthase kinase-3*β* gene transfer sustains apoptosis, inhibits proliferation of vascular smooth muscle cells, and reduces neointima formation after balloon injury in rats. *Arteriosclerosis, Thrombosis, and Vascular Biology*.

